# Contrasting Hygiene-Related Gastrointestinal Infections and Stress-Related Diseases at a Primary Health Care Facility within a Sub-Saharan African City: Before and during the COVID-19 Pandemic

**DOI:** 10.3390/diseases11010002

**Published:** 2022-12-22

**Authors:** Patience B. Tetteh-Quarcoo, Emmanuel Afutu, Madonna Wiafe-Ansong, Fleischer C. N. Kotey, Nicholas T. K. D. Dayie, Eric S. Donkor, John Ahenkorah, Emilia Asuquo Udofia, Patrick F. Ayeh-Kumi, Bartholomew Dzudzor, Isaac Julius Asiedu-Gyekye

**Affiliations:** 1 Department of Medical Microbiology, University of Ghana Medical School, Accra P.O. Box KB 4236, Ghana; 2 Department of Community Health, University of Ghana Medical School, Accra P.O. Box KB 4236, Ghana; 3 FleRhoLife Research Consult, Teshie, Accra P.O. Box TS 853, Ghana; 4 Department of Anatomy, University of Ghana Medical School, Accra P.O. Box KB 4236, Ghana; 5 Department of Medical Biochemistry, University of Ghana Medical School, Accra P.O. Box KB 4236, Ghana; 6 University of Ghana School of Pharmacy, College of Health Sciences, University of Ghana, Accra P.O. Box KB 52, Ghana

**Keywords:** COVID-19, stress-related diseases, gastrointestinal infections, depression

## Abstract

Background: With the advent of the COVID-19 pandemic caused by SARS-CoV-2, protocols such as social distancing and upscaling of hygiene practices were implemented to limit the spread of the disease. Meanwhile, along with COVID-19 came stress due to restrictions on movement, trade and transport, and closure of schools, among others. Aim: This study compared the prevalence of hygiene-related gastrointestinal infections and stress-related diseases before (March 2019–February 2020) and during (March 2020–February 2021) the COVID-19 pandemic. Methodology: This was a retrospective single-center review of deidentified patient data from the Korle Bu Polyclinic, Accra, Ghana. Results: Comparing the pre-COVID-19 era to the COVID-19 era, there was a statistically nonsignificant change in the number of cases and prevalence of gastroenteritis and enteric fever (*p* = 0.084 and 0.081, respectively), although for gastroenteritis, the prevalence was higher for the pre-COVID-19 era compared to during COVID-19 by 1.8 per 1000 cases, while that of enteric fever was higher during the COVID-19 era compared to the pre-COVID-19 era by 1.0 per 1000 cases. Of the stress-related diseases, statistically significant increases in the prevalence of anxiety disorders (*p* = 0.028), insomnia (*p* = 0.001), and headache (*p* = 0.010), were noted, with 2.3, 5.5, and 2.4 per 1000 cases, respectively. There were more female cases than male cases recorded for depression (*p* = 0.001), headache (*p* = 0.010), and hypertension (*p* = 0.001) during the pandemic, and these were statistically significant. Conclusion: During the pandemic, a significant increase in the prevalence of stress-related diseases was observed. However, a statistically nonsignificant change was recorded for gastrointestinal infections, with females reporting more of these disorders. Consequently, it is important to strengthen the capacity for managing stress-related conditions alongside diseases that cause pandemics when they arise.

## 1. Introduction

The World Health Organization (WHO) declared the SARS-CoV-2-caused coronavirus disease (COVID-19) a global pandemic on 11 March 2020 [1]. In Ghana, the first two COVID-19 cases were reported a day after this declaration [2]. In parallel, the Government of Ghana embarked on public education campaigns regarding preventive measures recommended by the WHO to reduce person-to-person transmission, with a concurrent herd effect on transmission of other pathogens [3,4]. These included avoiding or limiting physical contact (including handshakes and other forms of usual contact), regular handwashing with soap under running water, rubbing of hands with sanitizers possessing 70% alcohol strength, and reducing/limiting large gatherings (such as funerals, church activities, festivals and conferences) [5]. Similar to what was done elsewhere across the globe, educational institutions and borders were closed for a period of time and a partial lockdown was instituted in Accra and Kumasi, restricting movement and trade. This near-global lockdown was associated with an unprecedented drop in pollution [6].

As part of the local COVID-19-related remedial efforts, 137 markets were disinfected in the Greater Accra Region, along with several others in the Eastern, Northern, Northeast, and Savannah Regions [7]. These disinfection processes, together with the partial lockdown and other COVID-19 protocols, might have affected transmission of gastrointestinal and other pathogens.

Notwithstanding the gains chalked up from the elaborate interventions, ancillary effects on business activities, the global economy, food safety, health care, education, sports, and leisure have raised concerns about their potential composite impact on mental health [8,9]. For example, upon the onset of the pandemic, the Centers for Disease Control and Prevention (CDC) in 2021 warned that although social distancing is an important public health action to reduce the spread of the virus, it could cause feelings of isolation and loneliness that could increase stress and anxiety. Uncertainties about effective treatment for COVID-19, the large number of deaths, even in countries with high-quality health care services, and the impact on economies are also propagating factors of stress [9,10]. Chronic stress has been associated with an increased risk for development of diabetes, hypertension, and coronary artery disease [11,12]. In individuals with advanced atherosclerosis, short-term emotional stress may trigger cardiac events [13]. In spite of very little documented information on the impact of the pandemic on mental health in sub-Saharan Africa at the time, Semo and Frissa [14] predicted an immense impact on mental health unless sociocultural resilience factors and coping mechanisms were safeguarded. In light of this, stress-related conditions are likely to develop due to fear and anxiety of contracting COVID-19, closure of businesses, and restriction of trade and travel, among others.

The noncommunicable disease burden in sub-Saharan Africa has been slated to rise due to urbanization, population growth, and demographic transitions [15]; the incidence of gastrointestinal infections is also likely to be affected by the implemented COVID-19 protocols. Gastrointestinal infections contribute to the burden of infectious diseases worldwide and constitute the second-leading cause of preventable illness in children aged under 5 years [16]. In developing countries such as Ghana, poor hygiene, sanitation practices and poverty-related risk factors have contributed to this development. Evidence of a decrease in incidence will go to strengthen education on infectious disease transmission and the need to sustain hygiene-focused protocols long after COVID-19 is eliminated.

This study, therefore, retrospectively investigated differences in prevalence of gastrointestinal infections and stress-related diseases/disorders at a primary health facility in the capital city of Ghana before and during the COVID-19 pandemic. The impact of COVID-19 infection and its protocols on transmission has been assessed on some areas of health care [17,18,19,20,21,22,23,24,25]. However, to the best of our knowledge, this is the first study to have examined the differences in occurrence of both gastrointestinal infections and stress-related diseases/disorders at the same facility, before (pre) and during (after the advent of) the COVID-19 pandemic. It is noteworthy that although the setting for this study was a city, quite a number of the inhabitants have relatively low socioeconomic status.

We hypothesized that during the COVID-19 pandemic, measures such as avoiding or limiting physical contact (including handshakes and other forms of usual contact) and reducing/limiting large gatherings among the general population may lead to an increase in stress-related diseases/disorders, while regular handwashing with soap under running water and rubbing of hands with alcohol-based sanitizers could lead to a reduction in the prevalence of gastrointestinal infections reported at the facility investigated.

## 2. Materials and Methods

### 2.1. Study Design, Area, and Participants

This was a retrospective single-center review of de-identified data of patients (particularly, those who presented with gastrointestinal infections and stress-related diseases/disorders) from March 2019 to February 2021 at the Korle Bu Polyclinic. The polyclinic is located in the Ablekuma South sub-metropolitan area of the Accra Metropolis, Ghana. It is a 42-bed facility that offers primary 24 h inpatient and outpatient health care services (radiological, laboratory, pharmaceutical, and child welfare clinics, among others) to individuals who reside within the catchment area of the clinic [26]. 

Any period preceding 12 March 2020 (when the first COVID-19 cases were reported in the country) qualified to be part of the “pre-COVID-19 era” [2]. However, as the period covered in the analyzed dataset ends with February 2021, the “during COVID-19 era” in this study (March 2020 to February 2021) spanned one year. Hence the one-year period that preceded the first report of COVID-19 in Ghana (March 2019 to February 2020) was selected to represent the “pre-COVID-19 era”. This ensured equal timespans for the two periods under comparison—“pre-COVID-19 era” and “during COVID-19 era” (which we deemed a good design for such a research).

### 2.2. Keywords and Inclusion and Exclusion Criteria

In this study, hygiene-related gastrointestinal infections refer to infections of bacterial, viral or parasitic origin that cause inflammation of the gastrointestinal tract, i.e., the stomach, small intestines and the colon [27]. As noted by Liu et al. [28], stress is a state of threatened homeostasis provoked by a psychological, environmental, or physiological stressor. In line with this, in this study, stress-related diseases refer to health problems that could be associated with stress; these include anxiety disorders, insomnia, depression, headache, migraine and psychosis, and cardiovascular diseases (hypertension, hypertensive heart disease and angina). The choice of headache, migraine, and psychosis may not appear specific, since headache and migraine are multifactorial disorders that might not be strictly associated with stress-related conditions [29]. Therefore, the selected patients had their clinical records carefully reviewed to ensure that the headaches were not resultant of the commonly expected infectious causes of headache in the study setting, such as malaria and others, with at least one other stress-related clinical sign.

The study included data on patients who received at least one diagnosis of a gastrointestinal infection between March 2019 and February 2021 as well as those who received at least one diagnosis of a stress-related disease/disorder from a doctor at the facility during the same period. Data on patients who received the diagnosis of the above-named conditions outside the study facility were excluded, as were data involving incomplete patient records.

### 2.3. Statistical Analysis

The analyses were carried out using Microsoft Excel and Statistical Products and Services Solutions (SPSS) version 25. Quantitative data of patients were expressed as means ± standard deviation (SD). Descriptive analyses using percentages and frequencies were done and presented as line graphs and tables. Changes in occurrence of gastrointestinal infections and stress-related diseases/disorders were assessed by comparing the results from March 2019–February 2020 to those obtained for March 2020–February 2021, and the difference in prevalence between both periods was calculated. The proportions of men and women and age groups were compared using the independent-samples chi-squared test. Differences in variables whose *p* values were less than 0.05 were considered statistically significant.

### 2.4. Ethical Issues

Approval for the study was obtained from the Medical Directorate of the Korle Bu Teaching Hospital (KBTH-ADM/00039/2022), which has oversight of research conducted within the constituent units of the Korle Bu Teaching Hospital.

## 3. Results

### 3.1. Total Occurrence of Gastrointestinal and Stress-Related Cases

**Figure 1:**
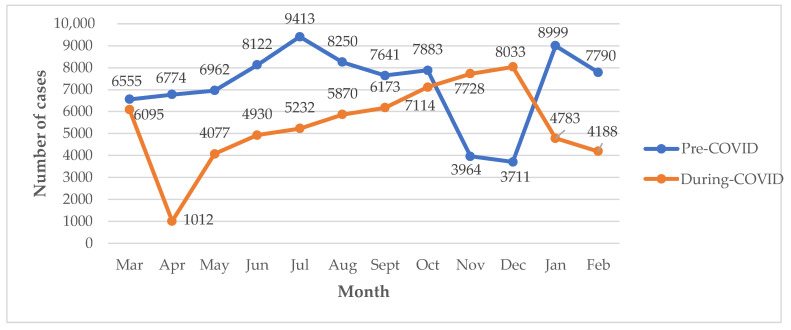
Trend of the total number of recorded cases at the hospital pre-COVID-19 and during COVID-19. Mar—March, Apr—April, Jun—June, Jul—July, Aug—August, Sept—September, Oct—October, Nov—November, Dec—December, Jan—January and Feb—February.

In the pre-COVID-19 era, the total number of cases was generally higher in the early part of the period, with the highest of the 9413 cases recorded in July 2019. In the latter part of the period, fewer cases were recorded, with a further rise towards the end (Figure 1). The lowest number of recorded cases was in December 2019 (Figure 1). During the COVID-19 era, the lowest number of cases was recorded in April 2020 (1012 cases), after which there was a steady rise, hitting 8033 cases in December 2020. A peak in the number of cases was observed in December 2020, after which there was a drop (Figure 1).

### 3.2. Cases of Gastrointestinal Infections

In the pre-COVID-19 era, the number of recorded gastroenteritis cases was higher than in the COVID-19 era in the earlier part of the year, with the highest number of cases recorded in June 2019 (Table 1). The number of cases was lower at the latter part of the year, with the lowest number recorded in October 2019 (82 cases) (Table 1). During the COVID-19 era, April 2020 saw a sharp decline in gastroenteritis cases and had the lowest number of recorded cases, after which the number of cases began to steadily rise until a peak was reached in January 2021 (Table 1). Although the prevalence of gastroenteritis was lower in March in the COVID-19 era than in the pre-COVID-19 era, it rose sharply in April, after which it dropped and remained lower than in the pre-COVID-19 era (Table 1). The prevalence, however, rose again sharply in January (Table 1).

The number of recorded enteric fever cases was generally higher in the earlier part of the pre-COVID-19 era, with the highest number recorded in July 2019 and the lowest in March 2019 (Table 1). During the COVID-19 era, the lowest number of cases was recorded in April 2020, while the highest was in December 2021 (Table 1). The prevalence of enteric fever fluctuated during the COVID-19 era, just as in the pre-COVID-19 era, but saw a reduction in April (Table 1). The prevalence, however, rose in July 2020, with a peak prevalence seen in February 2021 (Table 1).

### 3.3. Cases of Stress-Related Diseases/Disorders

The highest number of anxiety disorder cases recorded in the pre-COVID-19 era was in September 2019, with January and February 2020 recording an increase in the number of cases from the three months prior (Table 2). During the COVID-19 era, the highest number of cases was recorded in October 2020 and the lowest in April 2019 (Table 2). In all the months, the prevalence was higher during the COVID-19 era than in the pre-COVID-19 era (Table 2). The highest number of insomnia cases was recorded in January 2020 and the lowest in November 2019 in the pre-COVID-19 era (Table 2). During the COVID-19 era, the lowest was recorded in April 2020 and the highest in February 2021 (Table 2). The prevalence was higher in all the months during the COVID-19 era than in the pre-COVID-19 era (Table 2).

With respect to depression, the highest number of cases was observed in August 2019 in the pre-COVID-19 era, while the highest during the COVID-19 era was in June 2020 and January 2021 (Table 2). The lowest number of cases was recorded in November 2019 in the pre-COVID-19 era, while during the COVID-19 era, this occurred in March and April 2020 (Table 2). During the COVID-19 era, the prevalence of depression was higher in April, November and December 2020 and January and February 2021 than in those same months in the pre-COVID-19 era (Table 2). There was a reduction in prevalence in the other months.

Regarding headache, the highest number of cases was recorded in August 2019 and January and February 2021 in the pre-COVID-19 era (Table 3). During the COVID-19 era, a higher number of cases was recorded in the latter part of the period, the highest being in November 2020 (Table 3). Apart from March, April, May and August, the prevalence of headache was higher during the COVID-19 era than in those same months in the pre-COVID-19 era (Table 3).

The highest number of migraine cases in the pre-COVID-19 era was recorded in June 2019, although at the end of the period, there had also been a rise in the number of cases (Table 3). The lowest numbers were recorded in October, November and December 2019 in the pre-COVID-19 era (Table 3). During the COVID-19 era, October 2020 saw the highest number of recorded cases, the lowest being in April 2020 (Table 3). In March as well as August through to December 2020, the prevalence was higher during the COVID-19 era than in those same months in the pre-COVID-19 era (Table 3).

The highest number of psychosis cases recorded in the pre-COVID-19 and COVID-19 eras were in October 2019 and November 2020, respectively (Table 3). The lowest in the pre-COVID-19 era was in March 2019, while in the COVID-19 era, it was in March and April 2020. In April through to August 2020, the prevalence was higher than that recorded in the same set of months in the pre-COVID-19 era (Table 3). The same results were seen in February 2021. The other months recorded a lower prevalence during the COVID-19 era (Table 3).

### 3.4. Cases of Cardiovascular Diseases

Regarding hypertension, the number of cases dropped in the early parts of the pandemic, with the lowest recorded in April 2020, after which the number of cases rose, with concurrent fluctuations (Table 4). The highest number of cases during the COVID-19 era was recorded in December 2020 (Table 4). Generally, the numbers were lower during the pandemic than in the pre-pandemic era. Comparing both periods, the prevalence was generally lower during the COVID-19 era, but in May, August, January, and February, however, the prevalence was higher (Table 4).

With respect to hypertensive heart diseases, in the pre-COVID-19 era, the highest number of cases was recorded in October 2019 and the lowest in December 2019 (Table 4). During the COVID-19 era, the lowest recorded number was in April 2020 and the highest in December 2020 (Table 4). Comparing the two periods, the number of cases was generally lower in the latter part of the year, with exceptions being the numbers recorded for January and February 2021, although the difference was not much (Table 4). The prevalence was higher in March, April, May, July and the months following October during COVID-19 era than in the pre-COVID-19 era (Table 4).

As regards angina, there were fluctuations in the number of recorded cases in both the pre-COVID-19 and COVID-19 eras. The highest figures were recorded in October and September 2019 in the pre-COVID-19 era and during the COVID-19 era, respectively, while the lowest figures were recorded in March and April 2020, respectively (Table 4). The prevalence was higher at the beginning of the period during the COVID-19 era; it dropped in October and November, and rose in December, only to reduce again (Table 4).

### 3.5. Cerebrovascular Accident/Stroke

In the pre-COVID-19 era, the number of cases was higher in the earlier part, with a peak in July 2019, although it dropped in November 2019, the month in which the lowest number of cases was recorded (Table 5). During the COVID-19 era, the highest number was recorded in June 2020 and the lowest in April 2020 (Table 5). A higher prevalence was seen in the beginning and latter parts of the period during the COVID-19 era than in the pre-COVID-19 era, but from July to October during the COVID-19 era, the prevalence was lower (Table 5).

### 3.6. Differences in Prevalence between Pre-COVID-19 and COVID-19 Eras

Regarding gastroenteritis, there was a statistically nonsignificant change in the prevalence and total number of cases recorded, even though the number of cases recorded in the pre-COVID-19 era (*n* = 1343) was higher than during the COVID-19 era (*n* = 901). Similarly, a statistically nonsignificant change was observed for the prevalence and total number of cases of enteric fever recorded, despite a slightly higher number of cases observed during the COVID-19 era (*n* = 260) than in the pre-COVID-19 era (*n* = 256) (Table 6). In the case of anxiety disorders, insomnia, and headache, there were statistically significant increases in both the prevalence and total numbers of cases during the COVID-19 era compared to the pre-COVID-19 era (with *p* values of 0.028, 0.001 and 0.010, respectively). However, there was a statistically nonsignificant change in the prevalence and total number of angina cases recorded, even though more cases were recorded during the COVID-19 era (*n* = 92) than in the pre-COVID-19 era (*n* = 68) (Table 6). Concerning depression and hypertension, there was a statistically nonsignificant change in the number of cases and prevalence, although the number of cases for the pre-COVID-19 era was higher than that for the COVID-19 era (Table 6). Similarly, as regards migraine, hypertensive heart disease, and cerebrovascular accident (CVA)/stroke, a statistically nonsignificant change was observed, although more cases were recorded in the pre-COVID-19 era (Table 6).

### 3.7. Relationships between Age and Prevalence of the Diseases in the Pre-COVID-19 and COVID-19 Eras

The mean ages of the individuals presenting with all the diseases were lower during the COVID-19 era than in the pre-COVID-19 era, except for gastroenteritis and angina (Table 7). None of these changes were, however, statistically significant (Table 7).

### 3.8. Relationships between Gender and Prevalence of the Diseases in the Pre-COVID-19 and COVID-19 Eras

There were more female than male cases recorded for all the diseases in the pre-COVID-19 era, except for angina (Table 8). In the COVID-19 era, there were more female cases than male cases recorded for all the diseases, except for angina and stroke, with only the gender differences for depression, headache, and hypertension being statistically significant (Table 8).

## 4. Discussion

In a bid to limit the spread of the COVID-19 pandemic following its onset, various countries, including Ghana, implemented interventions such as upscaling of hand hygiene practices and education as well as restrictions on movement and social interactions. Although associated with a significant decrease in infectious disease morbidity, these alongside the stress induced by the pandemic may have resulted in an increase in mental health disorders, such as anxiety, depression, and other diseases that are exacerbated by stress [30]. This study investigated whether there was a change in the prevalence of gastrointestinal infections and stress-related diseases during the COVID-19 pandemic, using de-identified data obtained from the Korle Bu Polyclinic in Accra, Ghana.

Contrary to expectations that given the implemented WHO-recommended interventions [4], the prevalence of gastrointestinal infections may reduce in the COVID-19 era relative to the pre-COVID-19 era, the findings revealed the reduction to be statistically nonsignificant at the single-center studied. In contrast, a significantly lower gastrointestinal infection prevalence was reported by Amar et al. [30] and Tanislav and Kostev [31] for the COVID-19 era compared to the pre-COVID-19 era in their studies carried out in Israel and Germany, respectively. Ahn et al. [32], on the other hand, reported a non-significant decrease in the incidence of viral gastrointestinal infections. The hypothesis the researchers proffered for their observation was that while the transmission of viral gastrointestinal infections required a more direct contact, that of bacterial gastrointestinal infections were mainly through food [32].

The comparable gastrointestinal infection prevalence between the pre-COVID-19 and COVID-19 eras in the current study could be due to a number of reasons. First, in some parts of Ghana, adherence to COVID-19 protocols was poor, probably stemming from misconceptions that no case of COVID-19 infection had yet occurred in the country and/or that people from the tropics were immune to the virus [33,34]. Also possible is the contribution of the easing of restrictions towards the latter part of 2020, during which the euphoria associated with large gatherings may have caused laxity in adherence to the safety protocols. Moreover, the prevalent economic hardships and concurrent hikes in prices during the pandemic may have resulted in more individuals purchasing ready-to-eat foods from vendors, with little or no regard for the microbial safety of these foods, potentially predisposing the consumers to gastrointestinal infections [5]. Furthermore, difficulties in accessing hand sanitizers, face masks, and other items crucial to the prevention of COVID-19 transmission may have negatively impacted adherence. These factors may have, to varying degrees, blurred the behavioral adjustments that would have significantly driven down the gastrointestinal infection prevalence in the COVID-19 era.

The parallelism in the overall gastrointestinal infection prevalence notwithstanding, fewer cases of gastrointestinal infection were recorded early in the COVID-19 era relative to analogous periods in the pre-COVID-19 era. This observation could possibly be attributed to the implementation of measures recommended by the WHO to reduce person-to-person transmission [4]. Also, fewer cases recorded during the early part of the pandemic period than the pre-pandemic period may have been fueled by the fear that some probably entertained about contracting COVID-19 from hospital visits; such fears may have resulted from reports of COVID-19-positive cases involving personnel of some health care centers [2]. Conversely, the higher number of gastrointestinal infection cases recorded during the latter part of the COVID-19 era could be explained by the possibility that people might have relaxed in the adherence to the recommended safety protocols. In addition to these, some individuals may have felt more comfortable reporting to the hospital with gastrointestinal infections than with flulike symptoms, owing to the fear that they may potentially be quarantined should they be confirmed to be having COVID-19 infection. However, as explained earlier, the findings reported in this study originate from a single health care facility, warranting caution in their generalization to all health care facilities in the country or in other geographical locations.

The higher number of gastrointestinal infection cases involving females than males in both the pre-COVID-19 and COVID-19 eras could be due to the probability that females in the catchment area might have better health-seeking behavior than the males, leading to more frequent hospital visits, as has been reported elsewhere [35,36].

The statistically significant increase in the prevalence of anxiety disorders seems to support our hypothesis that the restrictive measures implemented during the COVID-19 era may lead to increased stress-related diseases/disorders. Likewise, Santabárbara et al. [37] and Santomauro et al. [38] demonstrated an increase in the proportions of individuals with anxiety disorders during the pandemic. The rapid spread of the virus, the high mortality rate associated with the disease, and fear of contracting the virus may have contributed to the increasing prevalence of anxiety disorders. It has been shown that exceptional situations like isolation can aggravate psychological disorders [39]. The feelings of isolation brought on by the restrictions placed on movement may have also worsened symptoms in those who already had anxiety disorders. This observation is noteworthy, since individuals with anxiety disorders experience significant impairment in how they function in their social, occupational, and physical domains [40], in such circumstances. The negative impacts of anxiety on various functional domains of life have been emphasized by earlier studies, eventually contributing to a poorer overall quality of life [41,42,43,44].

The lower mean age recorded for those reporting to the health care facility with anxiety disorders during the pandemic compared to that observed for the pre-pandemic period agrees with the study by Santomauro et al. [38], which estimated a higher prevalence of anxiety disorders among younger age groups than in the older ones due to closure of schools, restriction of social interactions, and economic crises brought on by the pandemic. In contrast, a study by Wang et al. [40] reported no association between age and stress levels, and hence anxiety. The break from school or work hectic schedules and the periods of rest that accompanied the social restrictions may have served as a relaxation period for some students and working adults. The observed higher number of anxiety cases involving females agrees with studies that have demonstrated a general predisposition of females to anxiety and other mental health conditions [36,45]. It is, however, in contrast to what was reported by Wang et al. [40]—that there was no association between gender and stress levels during the pandemic.

That insomnia prevalence significantly increased during the COVID-19 era agrees with what was reported in the study by Cénat et al. [41]—a higher prevalence of insomnia was reported among populations affected by COVID-19 than in the general population. The observed increase in prevalence has important clinical implications, since insomnia negatively affects the quality of life [46,47], which is further worsened by comorbid conditions [48,49]. Scalo et al. [50] reported that diagnosed insomnia was associated with consistent decreases in both physical and mental health-related quality of life scores. The increase in insomnia prevalence recorded in this study may have been as a result of fear of contracting the virus and the economic crisis that came with the pandemic [9]. A study conducted in Bangladesh by Hasan et al. [48], however, found that the prevalence of insomnia decreased during the second wave of the pandemic. The researchers attributed this to the fact that their target population may have adapted to the stressful pandemic situation, hence reducing their risk of mental health problems. That a higher number of the insomnia cases recorded during the pandemic involved females may be due to the higher vulnerability of females to mental health conditions compared to their male counterparts [51]. Females have also been reported to have higher knowledge and fear of the COVID-19 pandemic [48], and hence the increased anxiety. In contrast, a study by Pizzonia et al. [49] did not find any association between gender and insomnia symptoms. The lower mean age recorded for those reporting to the health care facility with anxiety during the pandemic compared to the pre-pandemic period may be due to the younger age groups being more stressed than the older.

The observed reduction in the prevalence of depression during the pandemic is contrary to what has been reported in other studies, and may be attributable to poor health seeking behavior, probably leading to less frequent hospital visits [38,44]. To illustrate, more individuals are expected to be depressed during the pandemic owing to the resultant minimal social interaction from the implemented restrictions [9]. Another possible reason for our observation could be that the break from work or school made the general population more relaxed, and hence less depressed.

Similar to the hypothesis provided for the higher number of anxiety cases involving females, that more of the depression cases occurring during the pandemic involved females could be attributed to the higher vulnerability of females to mental health conditions [51]. Additionally, females are more likely to report to the hospital with such symptoms than males [51]. That the prevalence of psychosis changed insignificantly during the COVID-19 era somewhat contrasts the report of O’Donoghue et al. [52] emanating from Australia. The researchers demonstrated an increase in first-episode psychosis during the pandemic, although not statistically significant [52]. Again, this change may have been due to increased relaxation time during the pandemic.

The significant increases in the prevalence of headaches and migraines (both of which can be triggered by stress) during the pandemic is consistent with what was reported by Tudor and Sova [53]. The researchers estimated an excess occurrence of headache of 4.53% relative to expected levels in normal times. Headaches have been found to both worsen quality of life for all age groups and place a significant burden on society [54]. According to Al-Hashel [55], among chronic pains experienced in childhood and adolescence, headaches are staggeringly the most prevalent, and can significantly lead to debilitated cognitive, emotional, and recreational functioning in all areas of life, ranging from homes to scholarly activities. Therefore, the observed significant increase in the prevalence of headache during COVID-19 period may have a critical clinical significance.

That the cases of headaches and migraines that were recorded during the pandemic involved individuals with a lower mean age is understandable, as individuals of the younger age groups are more likely to be stressed in the stressful pandemic situation [48,52]. Similarly, that more females accounted for the cases of headache than did males corroborates the findings of Wieckiewicz et al. [54], who reported a similar outcome. It is also possible that the longer periods that mothers spent caring for their children at home during the pandemic (because schools had been closed down) resulted in increased stress levels and to some extent accounted for the increased prevalence of headaches among the females.

Some studies have shown an elevation of blood pressure immediately after a disaster [56] due to the physical and mental stress these disasters induce. In the study of Elnaem et al. [57], 77% of hypertensive persons were reported to have had good blood pressure control during the pandemic. This is in line with the findings of this study, which showed a 3.3 per 1000 case reduction in hypertension cases recorded at the hospital. Apart from the likelihood that people were more relaxed because of time afforded them during lockdowns and closure of certain public places, those suffering from hypertension were more likely to have been adherent to their medications due to reports that individuals with co-morbidities had poorer prognosis when they acquired COVID-19. That more female cases of hypertension were recorded than were male cases in both pre-COVID-19 and COVID-19 eras is consistent with previous studies. For instance, the prevalence of hypertension has been reported to be higher in males than in females until menopause, after which there is a more rapid rise in women [58].

Regarding angina and stroke, the observed male preponderance is similar to what was found by Adeloye et al. [59]. Other studies have, however, not found a significant association between gender and cardiovascular disease and stroke [60,61]. Generally, though, the observed statistically nonsignificant changes in the prevalence of cardio- and cerebrovascular diseases like hypertensive heart disease, angina, and CVA/stroke during the COVID-19 era, as compared to the pre-COVID-19 era is at odds with previous reports [62,63]. Narita et al. [62] for instance, reported an increase in hypertension-related diseases immediately after a disaster until living conditions improved. Zhang et al. [63] also reported that anxiety status was associated with an increased risk of incident cardiovascular events during the COVID-19 pandemic. Interestingly, other studies reported a decline in stroke admission in centers all over the world [64,65]. This has been attributed to the possibility that those who had mild strokes decided to manage themselves at home instead of reporting to the hospital due to their fear of contracting the virus at the health care institutions [66]. Nonetheless, it is difficult to tell if this explanation specifically holds for the participants whose data were analyzed in this study. Indeed, the research findings are best interpreted in the context of the single facility studied. 

## 5. Conclusions

This study has shown anxiety disorders, insomnia and headache were more prevalent during the pandemic than in the pre-pandemic period. Despite the occurrence of fewer cases of gastrointestinal infection recorded early in the COVID-19 era relative to analogous periods in the pre-COVID-19 era, generally, a comparable prevalence of the infection was observed between the pre-COVID-19 and COVID-19 eras. More females reported to the hospital for headache and hypertension than did males, and this was attributed to better health-seeking behavior of females and them being more susceptible to stress than their male counterparts. Hospital attendance reduced during the first few months compared to the latter part. Although there were changes in the mean ages of those seeking care at the health facility between the pre-COVID-19 and COVID-19 eras, none of the changes was significant. More attention should be given to mental health conditions, especially during stressful disaster situations. The general population should be further educated about these conditions and hospitals alerted to prepare to handle them.

## 6. Limitations and Recommendations

This study had a few limitations. First, the gastroenteritis cases were not stratified into the type of the different infectious agents (such as viral, bacterial, parasitic, and fungal). It is, therefore, difficult to hypothesize if the results obtained were actually from infectious causes. Also, the study did not include patients who developed both gastrointestinal-related cases and stress-related diseases/disorders (i.e., common in both groups). However, this was a retrospective review of records focused on patients (particularly those who presented with either only gastrointestinal infections or stress-related diseases/disorders). We, therefore, recommend that future research may consider including such patients as their focus, to add insights among this group as well. Additionally, it is important to note that this was a single-center study (instead of a multicenter longitudinal study); therefore, conclusions from the present work cannot be generalized to the clinical situation in Ghana as a whole. Moreover, a study on association between occupation and stress levels during the pandemic could have helped determine if the observed changes in disease prevalence were due to stress of the pandemic or due to work. Furthermore, the current study was retrospective, and it is difficult to infer causality from the current set of results. Finally, the choice of some stress-related conditions, such as headache, migraine, and psychosis, may not present more specificity, since headaches and migraines are multifactorial disorders that might not be strictly associated with stress-related conditions. Future studies could further investigate other medical conditions that may have caused these cases.

## Figures and Tables

**Table 1:** Cases of gastroenteritis and enteric fever in the pre-COVID-19 and COVID-19 eras.

Months		Pre-COVID-19 Era (2019–2020)		COVID-19 Era (2020–2021)	
		Number of Cases	Prevalence	Number of Cases	Prevalence (Per 1000 Cases)
			(Per 1000 Cases)		
March	Gastroenteritis	125	19.1	76	12.5
	Enteric Fever	12	1.8	17	2.8
April	Gastroenteritis	103	15.2	22	21.7
	Enteric Fever	21	3.1	2	2
May	Gastroenteritis	125	18	38	9.3
	Enteric Fever	20	2.9	11	2.7
June	Gastroenteritis	145	17.9	45	9.1
	Enteric Fever	18	2.2	10	2
July	Gastroenteritis	134	14.2	64	12.2
	Enteric Fever	32	3.4	25	4.8
August	Gastroenteritis	142	17.2	73	12.4
	Enteric Fever	31	3.8	20	3.4
September	Gastroenteritis	127	16.6	70	11.3
	Enteric Fever	21	2.7	24	2.9
October	Gastroenteritis	82	10.4	89	12.5
	Enteric Fever	30	3.8	17	2.4
November	Gastroenteritis	93	23.7	92	12
	Enteric Fever	25	6.3	32	4.1
December	Gastroenteritis	107	28.8	117	14.6
	Enteric Fever	14	3.8	39	4.9
January	Gastroenteritis	87	9.7	141	29.5
	Enteric Fever	19	2.1	30	6.3
February	Gastroenteritis	72	9.2	73	17.4
	Enteric Fever	13	1.7	33	7.9

**Table 2:** Cases of anxiety disorders, insomnia, and depression in the pre-COVID-19 and COVID-19 eras.

Month		Pre-COVID-19 Era (2019–2020)		COVID-19 Era (2020–2021)	
		Number of Cases	Prevalence	Number of Cases	Prevalence
			(Per 1000 Cases)		(Per 1000 Cases)
March	Anxiety disorders	0	0	11	1.8
	Insomnia	20	3.1	22	3.6
	Depression	5	0.8	1	0.2
April	Anxiety disorders	0	0	5	4.9
	Insomnia	17	2.5	8	7.9
	Depression	6	0.9	1	1
May	Anxiety disorders	0	0	12	2.9
	Insomnia	11	1.6	39	9.6
	Depression	5	0.7	2	0.5
June	Anxiety disorders	12	1.5	14	2.8
	Insomnia	23	2.8	36	7.3
	Depression	11	1.4	6	1.2
July	Anxiety disorders	12	1.3	14	2.7
	Insomnia	14	1.5	49	9.4
	Depression	10	1.1	5	1
August	Anxiety disorders	8	1	24	4.1
	Insomnia	18	2.2	41	7
	Depression	15	1.8	2	0.3
September	Anxiety disorders	24	3.1	21	3.4
	Insomnia	18	2.4	56	9.1
	Depression	8	1	5	0.8
October	Anxiety disorders	0	0	31	4.4
	Insomnia	36	4.6	42	5.9
	Depression	6	0.8	2	0.3
November	Anxiety disorders	0	0	26	3.4
	Insomnia	8	2	48	6.2
	Depression	0	0	4	0.5
December	Anxiety disorders	0	0	25	3.1
	Insomnia	14	3.8	70	8.7
	Depression	1	0.3	4	0.5
January	Anxiety disorders	21	2.3	18	3.8
	Insomnia	42	4.7	54	11.3
	Depression	7	0.8	6	1.3
February	Anxiety disorders	20	2.6	21	5
	Insomnia	22	2.8	76	18.1
	Depression	6	0.8	5	1.2

**Table 3:** Cases of headache, migraine, and psychosis in the pre-COVID-19 and COVID-19 eras.

Month		Pre-COVID-19 Era (2019–2020)		COVID-19 Era (2020–2021)	
		Number of Cases	Prevalence (Per 1000 Cases)	Number of Cases	Prevalence (Per 1000 Cases)
March	Headache	47	7.2	27	4.4
	Migraine	11	1.7	12	2
	Psychosis	8	1.2	2	0.3
April	Headache	45	6.6	4	4
	Migraine	10	1.5	1	1
	Psychosis	1	0.1	2	2
May	Headache	54	7.8	15	3.7
	Migraine	14	2	7	1.7
	Psychosis	5	0.7	7	1.7
June	Headache	47	5.8	39	7.9
	Migraine	23	2.8	11	2.2
	Psychosis	7	0.9	8	1.6
July	Headache	51	5.4	34	6.5
	Migraine	16	1.7	8	1.5
	Psychosis	5	0.5	6	1.1
August	Headache	55	6.7	36	6.1
	Migraine	9	1.1	8	1.4
	Psychosis	12	1.5	10	1.7
September	Headache	32	4.2	47	7.6
	Migraine	15	2	19	3.1
	Psychosis	12	1.6	8	1.3
October	Headache	25	3.2	69	9.7
	Migraine	0	0	19	2.7
	Psychosis	21	2.7	6	0.8
November	Headache	17	4.3	72	9.3
	Migraine	0	0	20	2.6
	Psychosis	12	3	11	1.4
December	Headache	8	2.2	66	8.2
	Migraine	0	0	10	1.2
	Psychosis	7	1.9	8	1
January	Headache	55	6.1	60	12.5
	Migraine	21	2.3	9	1.9
	Psychosis	11	1.2	3	0.6
February	Headache	55	7.1	59	14.1
	Migraine	22	2.8	10	2.4
	Psychosis	5	0.6	4	1

**Table 4:** Cases of cardiovascular diseases (hypertension, hypertensive heart disease, and angina) recorded in the pre-COVID-19 and COVID-19 eras.

Month	Cases	Pre-COVID-19 Era (2019–2020)		COVID-19 Era (2020–2021)	
		Number of Cases	Prevalence (Per 1000 Cases)	Number of Cases	Prevalence (Per 1000 Cases)
March	Hypertension	1466	223.6	1206	197.9
	H. Heart Disease	72	11	84	13.8
	Angina	1	0.2	13	2.1
April	Hypertension	1498	221.1	197	194.7
	H. Heart Disease	83	12.3	26	25.7
	Angina	2	0.3	3	3
May	Hypertension	1589	228.2	994	243.8
	H. Heart Disease	70	10.1	43	10.5
	Angina	3	0.4	7	1.7
June	Hypertension	1626	200.2	962	195.1
	H. Heart Disease	92	11.4	56	11.3
	Angina	5	0.6	4	0.8
July	Hypertension	1815	192.8	962	183.9
	H. Heart Disease	90	9.6	74	14.1
	Angina	3	0.3	5	1
August	Hypertension	1593	193.1	1204	205.1
	H. Heart Disease	105	12.7	61	10.4
	Angina	7	0.8	8	1.4
September	Hypertension	1452	190	1161	188.1
	H. Heart Disease	97	12.8	79	12.7
	Angina	3	0.4	18	2.9
October	Hypertension	1649	209.2	1265	177.8
	H. Heart Disease	165	20.9	72	10.1
	Angina	14	1.8	6	0.8
November	Hypertension	814	205.3	1218	157.6
	H. Heart Disease	34	8.6	97	12.6
	Angina	5	1.3	5	0.6
December	Hypertension	858	231.2	1282	159.6
	H. Heart Disease	21	5.7	109	13.6
	Angina	5	1.3	13	1.6
January	Hypertension	1204	132.7	1194	251.7
	H. Heart Disease	80	8.9	73	15.3
	Angina	10	1.1	6	1.3
February	Hypertension	1531	196.5	1079	257.6
	H. Heart Disease	80	10.3	65	15.5
	Angina	10	1.3	4	1

H. Heart Disease—Hypertensive Heart Diseases.

**Table 5:** Occurrence of cerebrovascular accident/stroke cases in the pre-COVID-19 and COVID-19 eras.

Month	Pre-COVID-19 Era (2019–2020)		COVID-19 Era (2020–2021)	
	Number of Cases	Prevalence (Per 1000 Cases)	Number of Cases	Prevalence (Per 1000 Cases)
March	90	13.7	78	12.8
April	72	10.6	35	34.6
May	117	16.8	66	16.2
June	150	18.5	159	32.3
July	171	18.2	83	15.9
August	147	17.8	77	13.1
September	143	18.7	99	16.0
October	142	18.0	123	17.3
November	31	7.8	106	13.7
December	47	12.7	118	14.7
January	121	13.4	96	20.1
February	90	11.6	94	22.4

**Table 6:** Differences between the prevalence of the various diseases in the pre-COVID-19 and COVID-19 eras.

Disease/Condition	Pre-COVID-19 Era		COVID-19 Era		*p*-Value
	Number of Cases	Prevalence (Per 1000 Cases)	Number of Cases	Prevalence (Per 1000 Cases)	
Gastroenteritis	1343	15.6	901	13.8	0.084
Enteric Fever	256	3.0	260	4.0	0.081
Anxiety Disorders	97	1.1	222	3.4	0.028
Insomnia	243	2.8	541	8.3	0.001
Depression	80	0.9	43	0.7	0.132
Psychosis	106	1.2	75	1.1	0.528
Headache	491	5.7	528	8.1	0.010
Migraine	141	1.6	134	2.1	0.157
Hypertension	17,095	198.5	12,734	195.2	0.079
Hypertensive Heart Disease	989	11.5	839	12.9	0.261
Angina	68	0.8	92	1.4	0.055
CVA/Stroke	1321	15.3	1134	17.4	0.124

*p*-value of <0.05 was considered statistically significant. CVA—cerebrovascular accident.

**Table 7:** Relationships between age and prevalence of the diseases in the pre-COVID-19 and COVID-19 eras.

Disease/Condition	Mean Age ± SD		*p*-Value
	Pre-COVID-19 Era	COVID-19 Era	
Gastroenteritis	31.13 ± 23.325	34.63 ± 21.927	0.432
Enteric Fever	34.79 ± 19.425	40.77 ± 18.029	0.325
Anxiety Disorders	39.79 ± 15.890	36.01 ± 13.923	0.318
Insomnia	56.18 ± 17.853	54.70 ± 14.945	0.628
Depression	39.21 ± 14.908	34.67 ± 14.420	0.056
Psychosis	39.71 ± 15.193	33.15 ± 13.570	0.063
Headache	37.30 ± 16.640	36.82 ± 16.081	0.727
Migraine	33.07 ± 12.714	32.07 ± 11.059	0.521
Hypertension	60.01 ± 13.249	57.13 ± 14.302	0.376
Angina	43.60 ± 13.042	47.52 ± 12.421	0.126
CVA/Stroke	62.55 ± 14.379	61.22 ± 13.070	0.458

*p*-value of <0.05 was considered statistically significant. CVA—cerebrovascular accident.

**Table 8:** Relationships between gender and the prevalence of the diseases in the pre-COVID-19 and COVID-19 eras.

Disease/Condition	Gender	Frequency		*p*-Value
		Pre-COVID-19 Era	COVID-19 Era	
Gastroenteritis	Male	569 (42.4%)	417 (46.3%)	0.372
	Female	774 (57.6%)	484 (53.7%)	
Enteric Fever	Male	86 (33.6%)	115 (44.3%)	0.124
	Female	170 (66.4%)	145 (55.7%)	
Anxiety Disorders	Male	30 (30.9%)	91 (41.0%)	0.461
	Female	67 (69.1%)	131 (59.0%)	
Insomnia	Male	81 (33.3%)	202 (37.3%)	0.719
	Female	162 (66.7%)	339 (62.7%)	
Depression	Male	34 (42.9%)	11 (26.7%)	0.001
	Female	46 (57.1%)	32 (73.3%)	
Psychosis	Male	35 (33.3%)	35 (46.2%)	0.216
	Female	71 (66.7%)	40 (53.8%)	
Headache	Male	164 (33.3%)	149 (28.6%)	0.010
	Female	327 (66.7%)	379 (71.4%)	
Migraine	Male	45 (31.8%)	34 (25.5%)	0.065
	Female	96 (68.2%)	100 (74.5%)	
Hypertension	Male	4991 (29.2%)	4953 (38.9%)	0.001
	Female	12,104 (70.8%)	7781 (61.1%)	
Angina	Male	37 (55.0%)	60 (65.2%)	0.371
	Female	31 (45.0%)	32 (34.8%)	
CVA/Stroke	Male	624 (47.2%)	631 (55.6%)	0.513
	Female	697 (52.8%)	503 (44.4%)	

*p*-value of <0.05 was considered statistically significant. CVA—cerebrovascular accident.

## Data Availability

The data presented in this study are available on reasonable request from the corresponding authors: patborket2002@yahoo.com or pbtetteh-quarcoo@ug.edu.gh (P.B.T.-Q.); emmalineafutu@yahoo.com (E.A.); bdzudzor@ug.edu.gh (B.D.); Tel.: +233-244633251 (P.B.T.-Q.);+233-244202066 (E.A.); +233-205232611(B.D.).
